# Key biosynthetic gene subfamily recruited for pheromone production prior to the extensive radiation of Lepidoptera

**DOI:** 10.1186/1471-2148-8-270

**Published:** 2008-10-02

**Authors:** Marjorie A Liénard, Maria Strandh, Erik Hedenström, Tomas Johansson, Christer Löfstedt

**Affiliations:** 1 Chemical Ecology and Ecotoxicology, Department of Ecology, Lund University, Ecology Building, SE-22362, Lund, Sweden; 2 Microbial Ecology, Department of Ecology, Lund University, Ecology Building, SE-22362, Lund, Sweden; 3 Department of Natural Sciences, Institute of Natural Sciences, SE-85170, Sundsvall, Sweden

## Abstract

**Background:**

Moths have evolved highly successful mating systems, relying on species-specific mixtures of sex pheromone components for long-distance mate communication. Acyl-CoA desaturases are key enzymes in the biosynthesis of these compounds and to a large extent they account for the great diversity of pheromone structures in Lepidoptera. A novel desaturase gene subfamily that displays Δ11 catalytic activities has been highlighted to account for most of the unique pheromone signatures of the taxonomically advanced ditrysian species. To assess the mechanisms driving pheromone evolution, information is needed about the signalling machinery of primitive moths. The currant shoot borer, *Lampronia capitella*, is the sole reported primitive non-ditrysian moth known to use unsaturated fatty-acid derivatives as sex-pheromone. By combining biochemical and molecular approaches we elucidated the biosynthesis paths of its main pheromone component, the (*Z,Z*)-9,11-tetradecadien-1-ol and bring new insights into the time point of the recruitment of the key Δ11-desaturase gene subfamily in moth pheromone biosynthesis.

**Results:**

The reconstructed evolutionary tree of desaturases evidenced two ditrysian-specific lineages (the Δ11 and Δ9 (18C>16C)) to have orthologs in the primitive moth *L. capitella *despite being absent in Diptera and other insect genomes. Four acyl-CoA desaturase cDNAs were isolated from the pheromone gland, three of which are related to Δ9-desaturases whereas the fourth cDNA clusters with Δ11-desaturases. We demonstrated that this transcript (*Lca*-KPVQ) exclusively accounts for both steps of desaturation involved in pheromone biosynthesis. This enzyme possesses a Z11-desaturase activity that allows transforming the palmitate precursor (C16:0) into (*Z*)-11-hexadecenoic acid and the (*Z*)-9-tetradecenoic acid into the conjugated intermediate (*Z,Z*)-9,11-tetradecadienoic acid.

**Conclusion:**

The involvement of a single Z11-desaturase in pheromone biosynthesis of a non-ditrysian moth species, supports that the duplication event leading to the origin of the Lepidoptera-specific Δ11-desaturase gene subfamily took place before radiation of ditrysian moths and their divergence from other heteroneuran lineages. Our findings uncover that this novel class of enzymes affords complex combinations of unique unsaturated fatty acyl-moieties of variable chain-lengths, regio- and stereo-specificities since early in moth history and contributes a notable innovation in the early evolution of moth-pheromones.

## Background

**Figure 1:**
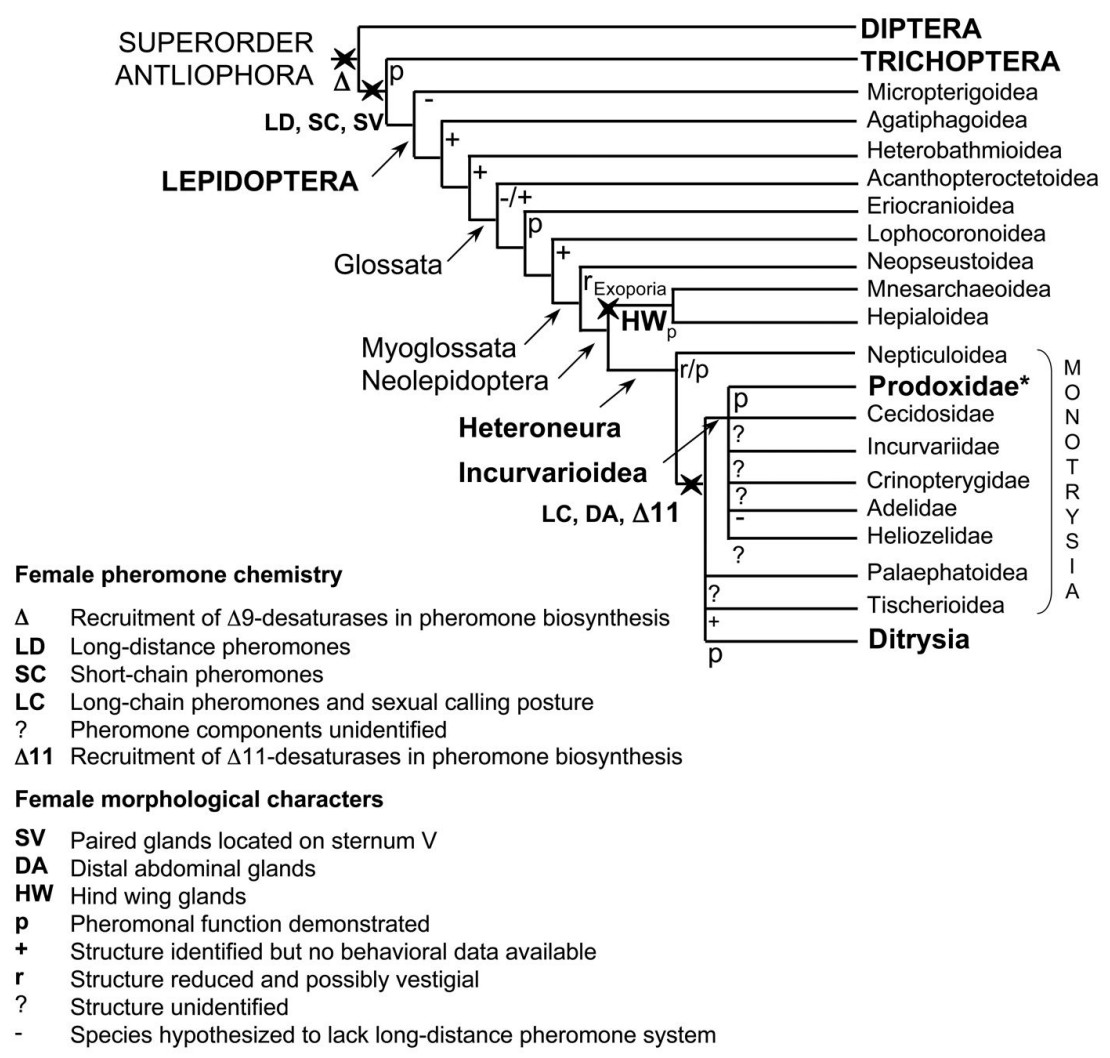
**Cladogram of the major lineages of the Lepidoptera and their relationships with Trichoptera and Diptera**. The sequence of lineages is adapted from [36-38], with information regarding the characters related to the evolution of female morphology and pheromone chemistry. Monotrysian and ditrysian species have one or two female genital opening(s), devoted to mating and/or oviposition, respectively [1]. The taxonomic position of *Lampronia capitella *is indicated with an asterisk (*).

The Lepidoptera, comprising the moths and butterflies [1], represent the second largest lineage of plant-feeding organisms and among all insect orders they seem to have radiated most recently [2]. Moths, including 95% of the extant lepidopteran species have evolved an efficient mate-communication system based on volatile sex pheromones produced by females. The vast majority of the higher Lepidoptera -the so-called Ditrysia (Fig 1)- uses unique blends of long-chain unsaturated alcohols, acetates or aldehydes in order to attract conspecific males over long distances [3,4]. These pheromones are produced in specialized pheromone glands located along the intersegmental membrane between the terminal abdominal segments VIII and IX [5]. These pheromone compounds are biosynthesized from saturated fatty-acid precursors along pathways involving a few discrete enzymatic reactions including the introduction of double bonds by specific desaturases, limited chain-shortening reactions and the formation of an oxygenated functional group (*e.g*., [6-9]).

An important step in insect pheromone biosynthesis is the involvement of specific desaturases introducing double bonds at specific positions in the fatty-acyl chain by removing two hydrogen atoms [7-10]. Insect desaturases are homologous to the ancestral Δ9 acyl-CoA desaturases of plants, vertebrates and fungi and are functioning as part of a multienzyme complex residing in the endoplasmic reticulum (ER) [11-14]. Certain moth desaturase lineages are believed to have arisen subsequently to the divergence of Lepidoptera and Diptera that took place around 300 million years ago (MYA) [10,15] and may have played a significant role in the evolution of sex-pheromones in Lepidoptera. Interestingly, the biosynthesis of many ditrysian oxygenated pheromones proceeds with various Δ11-desaturation reactions [7,15,16]. Up to this date desaturase-encoding genes have been investigated in a dozen ditrysian moths, depicting examples of elaborate evolution in which a minimal number of enzymes account for the chemical diversity and species-specificity of pheromone components found among different species [17-32].

Only a few primitive moth species have so far been investigated considering their chemical communication. Their pheromones are made of short-chain alcohols and ketones and release through sternal globular glands whose openings are located on the V^th ^abdominal segment [33-35]. The modern distal female abdominal gland and the typical ditrysian calling postures likely arose prior to divergence of the Ditrysia as a few examples are known in some monotrysian Heteroneura [reviewed in [36] and [37]]. However, the exact localization of distal gland producing-pheromone in non-ditrysian Heteroneura remains to be determined. Likewise, the origin of the ditrysian sex-pheromone, *i.e*., the emergence of mating-signals derived from saturated long-chain fatty acids and involving genes (*e.g*., desaturases) specific for pheromone production, remains obscure although it has been suggested to originate in a moth lineage prior to the divergence of Ditrysia and the other Heteroneura lineages [36,38] (Fig. 1). Recently, long-chain unsaturated pheromone components were identified from the abdominal tip in a moth species outside the Ditrysia; the sex pheromone of the currant shoot borer, *Lampronia capitella *(Incurvarioidea: Prodoxidae), was characterized as a mixture of (*Z,Z*)-9,11-tetradecadienol, and the corresponding acetate and aldehyde [37]. This monotrysian species constitutes the most primitive moth for which long-chain unsaturated pheromone components have been evidenced and provides a unique opening for investigating evolutionary aspects of the ditrysian pheromone mating-signals.

In this study we report on pheromone-gland precursor identification, molecular characterization and functional expression of desaturase transcripts from the pheromone gland of *L. capitella*, of which a functional Δ11-desaturase transcript that exhibits all of the required biochemical activities to biosynthesize the conjugated chemical structures of the major sex pheromone component. Our findings evidence that the novel desaturase lineages, (Δ9 (18C>16C) and Δ11) evolved before the split between the ditrysian and other heteroneuran moth lineages. By extension, our findings suggest that the specific biosynthetic functions inherited from early moth history and which contributed to structural variations in mating-signals may have played a role in the radiation of the higher Lepidoptera.

## Results

### Fatty-acid pheromone precursor identification

**Figure 2:**
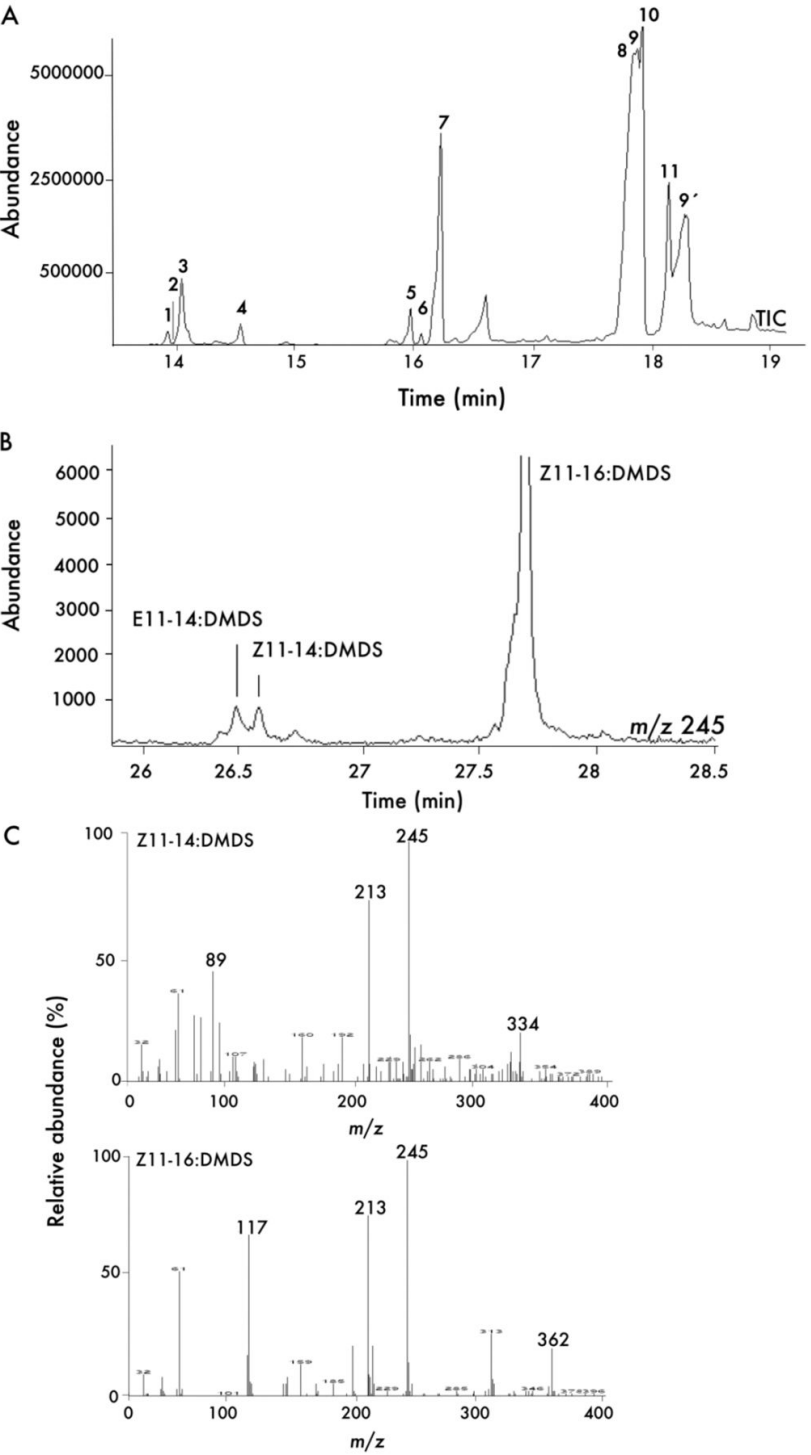
**GC-MS analyses of fatty-acid precursors from *Lampronia capitella *female abdominal tip extracts**. (**A**) Total ion current (TIC) chromatogram of a base methanolyzed abdominal tip lipid extract. Peaks are indicating: (1) Z9–14:Me; (2) E11–14:Me and Z11–14:Me; (3) 14:Me; (4) Z9,Z11–14:Me; (5) Z9–16:Me; (6) Z11–16:Me; (7) 16:Me; (8) Z9,Z12–18:Me; (9) Z9,Z12,Z15–18:Me; (10) Z9–18:Me; (11) 18:Me; (9') Z9,Z12,Z15–18:acid. (**B**) DMDS derivatives from a methanolyzed abdominal tip extract from *L. capitella*. The trace chromatogram is obtained by selection of the Δ11 diagnostic fragment at *m/z *245. Peaks labelled as Z11–14:DMDS, E11–14:DMDS and Z11–16:DMDS correspond respectively to the dimethyldisulfide adducts of methyl (*Z*)-11-tetradecenoate; of methyl (*E*)-11-tetradecenoate and of methyl (*Z*)-11-hexadecenoate. (**C**) Mass spectra of the Z11–16:DMDS and Z11–14:DMDS adducts confirming the double bond positions. The E11–14:DMDS exhibited a mass spectrum identical to the Z11–14:DMDS.

Methanolyzed samples of abdominal tips from *L. capitella *were prepared and analysed by GC-MS. In addition to the saturated fatty-acid methyl esters from C8 to C20 and the common unsaturated esters Z9–18:Me; Z9,Z12–18:Me; Z9,Z12,Z15–18:Me and Z9–16:Me, the esters identified also included Z9–14:Me, Z11–14:Me, E11–14:Me, Z11–16:Me and a methyl tetradecadienoate (Fig 2). Z9–12:Me was not detected either in FAME- or in DMDS-adduct analyses. Monounsaturated methyl esters exhibited the expected diagnostic ions at *m/z *74, M^+^, M^+ ^-31, M^+ ^-32, M^+^-74 (C16:1 = 268, 237, 236, 194; C14:1 = 240, 209, 208, 166, respectively) at expected retention times. GC-MS analyses of DMDS derivatives of a methanolyzed sample confirmed the identification of Z9-monoenes by detection of the characteristic ion at *m/z *217 at expected retention times (data not shown). DMDS adducts of the Z11–16:Me, Z11–14:Me and E11–14:Me exhibited the characteristic ion at *m/z *245 as shown in Fig. 2.

The double-bond positions of the methyl tetradecadienoate were identified as 9,11 by analysis of its MTAD derivatives that exhibited the diagnostic ions at *m/z *351 (M^+^), 194 and 322. The presence of a methyl 11,13-hexadecadienoate was also found (data not shown), exhibiting diagnostic MTAD adducts at *m/z *379 (M^+^), 194 and 350, which can be rationalised to be formed by chain-elongation of the relatively abundant Z9,Z11–14:acyl.

When the glands were incubated with D_9_-Z11–16:acid for 24 hours *in vivo*, the label was incorporated into Z9–14:Me (data not shown), a potential intermediate in pheromone biosynthesis, thereby indicating that Z9–14:acyl could be produced by β-oxidation of Z11–16:acyl.

### Characterization of desaturase transcripts

Total RNA was isolated from female abdominal tips of *L. capitella*. In PCR reactions using the corresponding cDNA as template and primers targeting conserved desaturase motifs [21], 550-bp DNA fragments were amplified that encompassed the central region of a desaturase gene. From the DNA information provided by the central region, four distinct transcripts were identified and their full-length cDNA sequences subsequently cloned.

The first desaturase transcript, *Lca*-QPAQ spans 1,427 bp and encompasses an ORF encoding a protein of 353 aa residues, which shares high aa sequence similarity with known Z9-desaturases (16C>18C), *e.g*., 81% with the Z9-desaturase from *Choristoneura parallela *[GenBank:AAN39701] and 80% with the Z9-desaturase from *Epiphyas postvittana *[GenBank:AAL35750].

The second desaturase transcript, *Lca*-SPVE spans 1,953 bp and contains an ORF encoding a 352-aa residue protein. This deduced protein shows 64% sequence similarity to *Lca*-QPAQ but higher similarity to other Z9-desaturases (18C>16C), *e.g*., 77% aa-sequence similarity to the Z9-desaturase from *Trichoplusia ni *[GenBank:AAB92583] and 76% to the Z9-desaturase from *Spodoptera littoralis *[GenBank:AAQ74257].

The third desaturase transcript, *Lca*-GATD spans 1,852 bp and encompasses an ORF encoding a 380-aa residue protein, which displays 51% sequence similarity to both *Lca*-QPAQ and *Lca*-SPVE and even higher similarity (73%) to the Z9-desaturase (C14-C26) from *C. parallela *[GenBank:AAQ12887].

**Figure 3:**
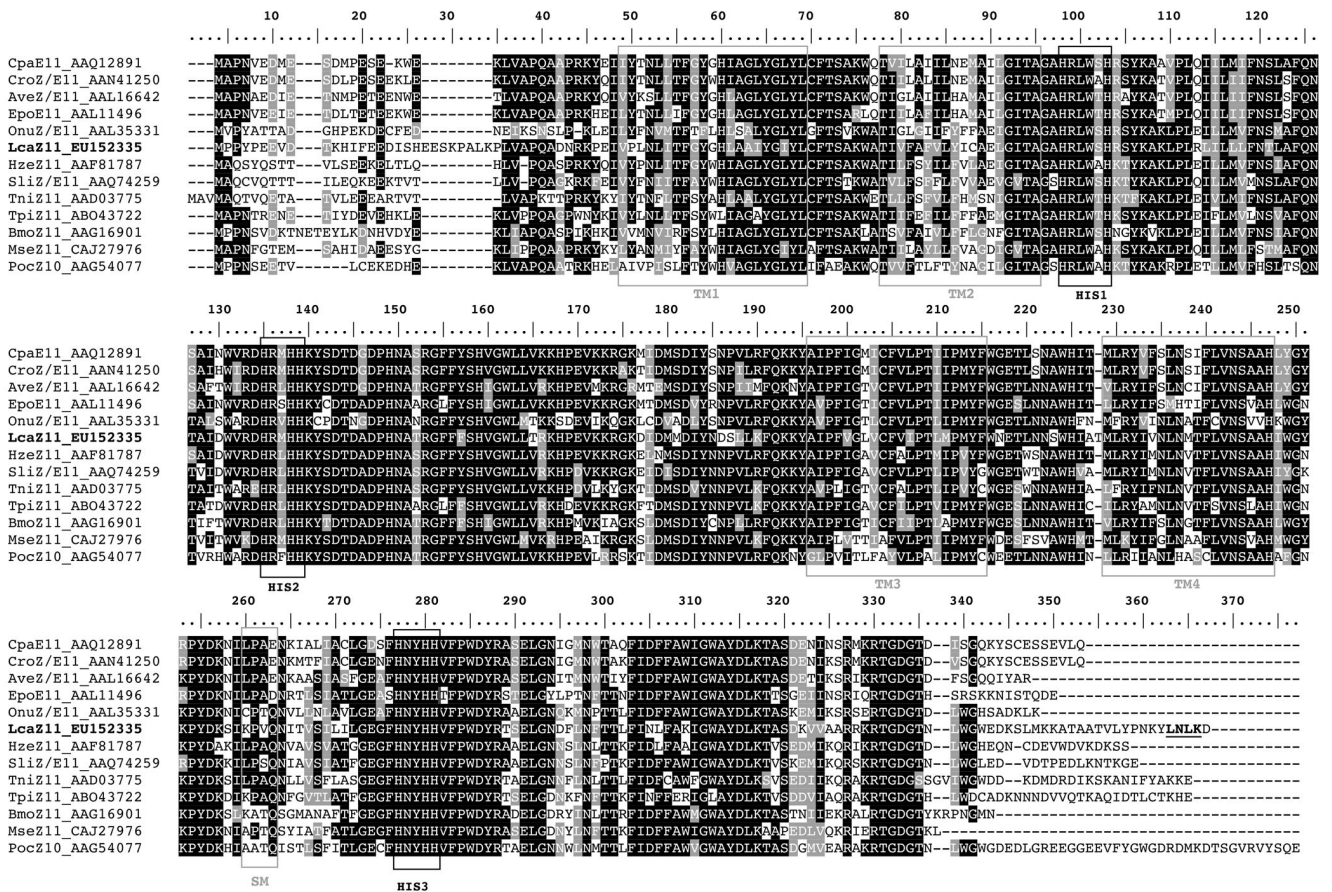
**Amino-acid sequence comparison of acyl-CoA desaturases belonging to the Δ11-lepidopteran desaturase subfamily**. The origin is indicated in the sequence according to: Cpa, *Choristoneura parallela *[25]; Cro, *Choristoneura rosaceanea *[20]; Ave, *Argyrotaenia velutinana *[23]; Epo, *Epiphyas postvittana *[24]; Onu, *Ostrinia nubilalis *[21]; Lca, *Lampronia capitella *(this study); Hze, *Helicoverpa zea *[18]; Sli, *Spodoptera littoralis *[28,29]; Tni, *Trichoplusia ni *[17]; Tpi, Thaumetopoea pityocampa [30]; Bmo, *Bombyx mori *[27]; Mse, *Manduca sexta *[31] and Poc, *Planotortrix octo *[19]. The sequence name also refers to the biochemical activity as well as the GenBank accession number (*Lca*-KPVQ is referred to as *Lca*Z11-EU152335). Black and grey backgrounds indicate aa identities and conservative substitutions, respectively. Boxed regions indicate the three conserved HIS domains of desaturases, the four protein transmembrane domains (TM1 to TM4) and the Δ11-desaturase signature motif (SM), respectively [15]. The proposed ER retention signal [61] is underlined and marked in bold face (aa positions 356–359).

**Figure 4:**
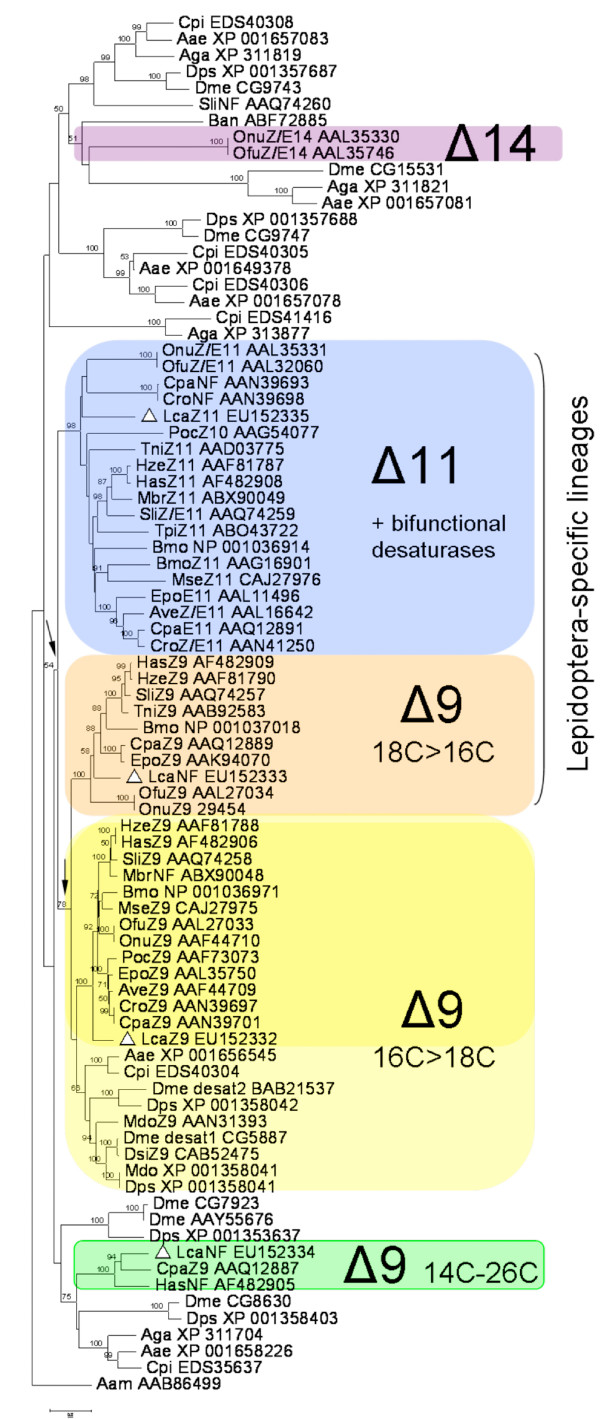
**Evolutionary tree of lepidopteran and dipteran desaturase genes**. Only genes from species for which complete sequences and biochemical activity have been reported were used in the tree construction, as well as predicted full-length sequences from dipteran genomes. The Neighbour-Joining tree was reconstructed using aa sequences and the JTT algorithm (MEGA 3.1, [63]); numbers along branches indicate bootstrap support from 1,500 replicates. The accession numbers for all sequences are given in connection with the abbreviated species name (and are listed in Fig. 3, in addition to Aae, *Aedes aegypti*; Aga, *Anopheles gambiae*; Ban, *Belgica antartica*; Cpi, *Culex pipiens*; Dme, *Drosophila melanogaster*; Dps, *Drosophila pseudoobscura*; Dsi, *Drosophila simulans *and Mdo, *Musca domestica *(Diptera) and Has, *Helicoverpa assulta*; Ofu, *Ostrinia furnacalis*, Mbr, *Mamestra brassicae *(Lepidoptera). The four desaturase-encoding cDNAs from this study are indicated by a triangle (Δ). Arrows indicate duplication events leading to lepidopteran-specific desaturase gene lineages. Biochemical activities are indicated in connection to the species name, NF refers to an non-functional transcript. The tree was rooted using the aa desaturase sequence from the tick *Amblyomma americanum *[10,21,30].

The fourth desaturase transcript, *Lca*-KPVQ, spans 1,671 bp and encompasses and ORF encoding a 360-aa residue protein. The deduced aa sequence shows 58%, 54% and 46% similarity to *Lca*-QPAQ, *Lca*-SPVE and *Lca*-GATD, respectively and shows between 60–65% similarity to various 11-insect desaturases (Fig. 3 and 4).

### Functional assay of Δ9-desaturases by complementation in mutant yeast using the expression vector YEpOLEX

A desaturase-deficient yeast cell line (*ole1*) was transformed with YEpOLEX plasmids containing the *Lca*-QPAQ, *Lca*-SPVE or *Lca*-GATD ORF, respectively. YEpOLEX-*Lca*-QPAQ transformants were able to grow on medium lacking supplemental unsaturated fatty-acids (UFAs), indicating that the *Lca*-QPAQ cDNA was encoding a desaturase that complemented the UFA auxotrophic *ole1 *strain. In contrast, YEpOLEX-*Lca*-SPVE and YEpOLEX-*Lca*-GATD transformants were unable to grow on media lacking UFAs. These results were confirmed using distinct verified gene constructs under identical experimental conditions. Chromatograms of methylated fatty-acid extracts from the *Lca*-QPAQ transformants showed three peaks with retention times corresponding to Z9–14:Me, Z9–16:Me and Z9–18:Me, in a 2:62:36 ratio. Fatty-acid methyl esters from yeast transformed with the Z9-desaturase gene of *H. assulta *(YEpOLEX-*Hass*-KPSE, [26]) were also prepared for use as a positive control. In this case the transformation resulted in the production of Z9–16:Me and Z9–18:Me, with a preference for palmitic acid, as shown in GC-MS analyses (data not presented). Thus *Lca*-QPAQ and *Hass*-KPSE are encoding Z9-desaturases displaying a substrate preference for palmitic acid, which is consistent as these desaturases are phylogenetically closely related (Δ9 16C>18C cluster) (Fig. 4). Double-bond position in the reaction products was confirmed by analyses of DMDS derivatives, which exhibited the characteristic fragment at *m/z *217 at expected retention times (data not shown). No distinctive products were detected in FAME or MTAD analyses after incubation of yeasts transformed with any of the YEpOLEX-*Lca*-constructs in the presence of Z11–14:Me.

### Functional assay of a Δ11-desaturase by complementation in mutant yeast using the expression vector pYEX-CHT

**Figure 5:**
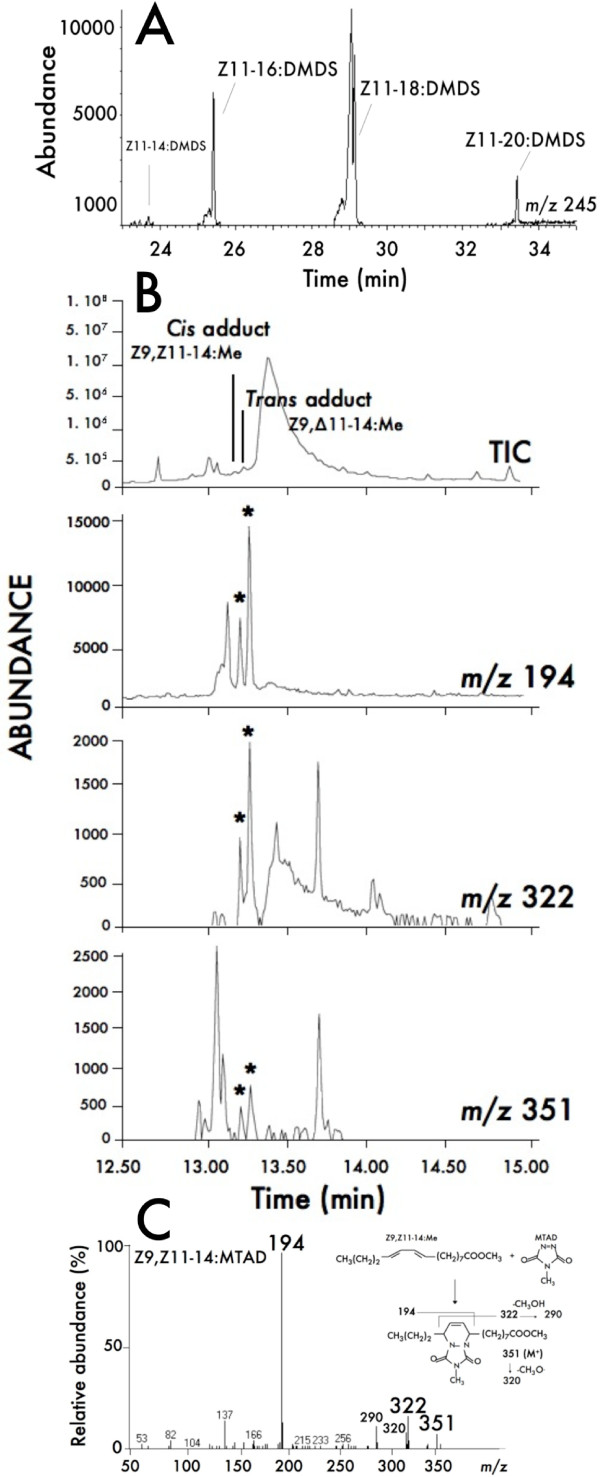
**GC/MS analyses of lipid extracts from *ole1 elo1 *yeast transformed with the *Lca*-Z11-KPVQ desaturase gene**. **(A) **DMDS derivatives from a methanolyzed lipid extract from yeast transformed with pYEX-*Lca*-Z11-KPVQ and grown in presence of 1 mM CuSO4. The chromatogram trace corresponds to the ion current obtained by extraction of the characteristic Δ11 DMDS fragments at *m/z *245. Peaks labelled as Z11–14:DMDS, Z11–16:DMDS, Z11–18:DMDS and Z11–20:DMDS correspond respectively to the dimethyldisulfide adducts of methyl (*Z*)-11-tetradecenoate, of methyl (*Z*)-11-hexadecenoate, of methyl (*Z*)-11-octadecenoate and of methyl (*Z*)-11-eicosenoate. **(B) **Analysis of MTAD derivatives of methyl dienoates from yeast transformed with pYEX-*Lca*^-^Z11-KPVQ and grown in presence of 1 mM CuSO_4 _and 0.5 mM Z9–14:Me. The top panel represents the total ion current (TIC) chromatogram. Peaks labelled as *cis *and *trans *adducts are produced upon MTAD reaction with methyl (*Z*,*Z*)-9,11–14-tetradecadienoate and methyl (*Z*,*Δ*)-9,11-tetradecadienoates, respectively. The delta symbol (Δ) refers to both *Z *or *E *geometrical configurations. The chromatogram traces in the lower panel are obtained by extraction of the characteristic ions at *m/z *194, 322 and 351, respectively. (**C**) Mass spectrum of MTAD adducts of methyl (*Z*,*Z*)-9,11-tetradecadienoate (Z9,Z11–14:MTAD) and methyl (*Z*,*Δ*)-9,11-tetradecadienoates (Z9,Δ11–14:MTAD) confirming the double-bond positions. MTAD-adducts of synthetic dienes have the same retention time and mass spectra than the natural compounds. Experiments were performed as described in the experimental section.

Since the *Lca*-KPVQ transcript could not be expressed in the pYES2.1 system (Invitrogen) neither in the *InvSc1 *nor in the *ole1 *yeast strains, the *Lca*-KPVQ ORF was ligated into a copper-inducible pYEX expression vector [39] to assess its desaturase activity and then transformed into a desaturase- and elongase-deficient mutant *ole1 elo1 *yeast strain [40]. GC-MS analyses of fatty-acid methyl esters from a recombinant yeast cell line grown in presence of Cu^2+ ^showed that the encoded protein produced a broad range of monounsaturated products. Transformed cells produced Z11–14:Me, Z11–16:Me, Z11–18:Me and Z11–20:Me in a 1:48:36:15 ratio. GC-MS analyses of DMDS derivatives prepared from the yeast transformants exhibited the characteristic fragment for a double-bond position between C11-C12 at *m/z *245 (Fig. 5A). All monoene products showed Z11 stereospecifity and no E11 isomers were found among DMDS derivatives. Besides Z11-monounsaturated acid products, cells supplemented with Z9–14:Me had produced a conjugated C14 dienoate as further confirmed by analyses of MTAD derivatives (Fig. 5B). GC-MS analyses of MTAD-derivatized samples, corroborated by injection of MTAD-derivatized synthetic standards, showed that both *cis *and *trans *MTAD adducts were produced by *Lca*-KPVQ yeast transformants. The two C14-MTAD adducts displayed identical diagnostic ions at *m/z *351 (M^+^), *m/z *322 and *m/z *194 (base peak) and different retention times (13.23 min and 13.29 min, respectively) (Fig. 5B–C). The presence of a *cis *adduct indicated that yeast cells supplemented with Z9–14:Me and transformed with *Lca*-KPVQ produced the (*Z,Z*)-9,11-tetradecadienoate. The *trans *adduct can be produced from both *Z,Z *and *Z,E *dienes [41], which does not allow to preclude the production of the *Z,E *diene. Conjugated 8,10-dienes (diagnostic adduct ion: *m/z *308) that were also detected in analyses of MTAD-derivatives (not shown) are an artefact of the MTAD reaction that takes place upon derivatization of 9-monoenes (*i.e*., Z9–14:Me) (A. Svatoš, pers. com.).

## Discussion

### Desaturases as key factor for pheromone evolution

There is no single explanation for the evolutionary and ecological success of Lepidoptera but it is rather thought to be the result of a cascade of successful evolutionary innovations [2]. The recruitment of certain lineages of desaturase genes to serve in pheromone biosynthesis might be one of these innovations that have played a significant role in the evolution of Lepidoptera, setting the scene for the development of a very effective mate-communication system [10,38]. The radiation of the Lepidoptera took off about 125 MYA, approximately at the time when the Ditrysia diverged from other heteroneuran Lepidoptera [2]. This evolutionary event appears to coincide with the emergence of a novel pheromone communication system, characterized by the involvement of novel desaturases in the production of long-chain unsaturated fatty-acid derivatives as pheromone components. In particular, Δ11-desaturases contributed significantly to the biosynthesis of a broad range of unsaturated pheromone components in ditrysian species and strongly increased the structural diversity of pheromone precursors [6-8]. In this study we demonstrate the involvement of a Δ11-desaturase in the pheromone biosynthesis in a non-ditrysian moth, *Lampronia capitella *(Incurvarioidea, Prodoxidae), which strongly supports the interpretation that the recruitment of the specialized desaturases to serve in the production of moth pheromone components occurred in the early evolution of Lepidoptera, before their extensive radiation.

### Involvement of a Δ11-desaturase gene in mate signalling in the primitive monotrysian moth L. capitella

**Figure 6:**
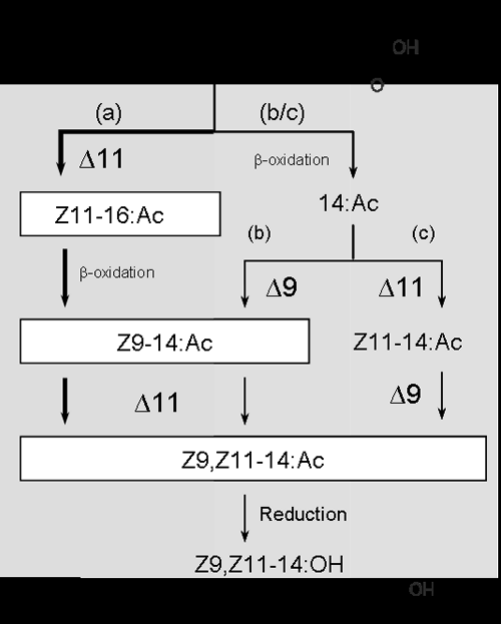
**The potential biosynthetic pathways leading to the major sex pheromone component, the (*Z,Z*)-9,11–14:OH, of the primitive currant shoot borer, *Lampronia capitella***. The pathway ***a***, which involves two successive Δ11-desaturations of acyl intermediates, is evidenced to be the major biosynthetic route in *L. capitella*.

We explored the potential pathways leading to the production of Z9,Z11–14:OH and the corresponding aldehyde and acetate, which are known pheromone components in *L. capitella *(Fig. 6). The absence of Z9–12:acyl excluded the possibility of having two successive interactions of a Δ9-desaturase in the pheromone biosynthesis and the observed fatty-acid profile was found compatible with all three hypothetical pathways shown in Fig. 6. Due to the occurrence of relatively large amounts of Z11–16:acyl in the gland and the confirmed possible chain-shortening of this intermediate to Z9–14:acyl, a scenario was eligible (pathway ***a ***in Fig. 6) that involves Δ11-desaturation of palmitic acid as an initial step in pheromone biosynthesis then followed by Δ11-desaturation of Z9–14:acyl.

Four full-length desaturase encoding cDNAs were characterized from the *L. capitella *abdominal tip and their deduced amino acid sequences notably revealed the three histidine-rich motifs (HIS boxes) that are characteristic of membrane-bound desaturases [13]. The *Lca*-QPAQ, *Lca*-SPVE and *Lca*-GATD transcripts shared high homologies with known Δ9-desaturases while the fourth transcript (*Lca*-KPVQ) showed a high amino acid sequence similarity with several Δ10, Δ11 and bifunctional desaturases from ditrysian moth species. Sequence analysis evidenced that the isolated transcript represented a typical fatty-acyl desaturase gene. Its predicted primary protein structure (Fig. 3) shared several key features with other known insect desaturases, like the position and the length of the transmembrane domains relative to the conserved HIS boxes and the xxxQ signature motif according to a desaturase-nomenclature previously proposed [15]. A reconstruction of the evolutionary history of desaturase genes (Fig. 4) finally indicated this candidate desaturase to cluster with members of the Δ11-desaturase lineage.

Functional characterization of the *Lca*-KPVQ gene product in a desaturase- and elongase-deficient yeast strain was performed and analyses of the unsaturated fatty-acid profiles of pYEX-*Lca*-KPVQ-expressing transformants established that the gene product displayed a Z11-desaturase activity with relatively broad substrate specificity. Saturated substrates from C8 to C22 occur naturally in the *ole 1 elo1 *yeast strain and the gene-product catalysed the removal of two hydrogen atoms from C14, C16, C18 and C20, with a strong substrate preference for palmitic acid (C16) as shown in Fig. 5A. This enzyme is also the first insect Δ11-desaturase reported to act on the C20:acyl substrate. After complete GC-MS analysis of both yeast methanolyzed extracts and their DMDS derivatives, the *Z *configuration of the double bond was confirmed in all newly formed unsaturated substrates and no *E *isomers were detected. Two Δ11-desaturases (*Ostrinia *sp., GenBank acc nos. AAL32060 and AAL35331) were characterized that produced *Z*-isomers of C16 and C18 acyl precursors in addition to *Z *and *E *isomers of C14 acyl precursors that were later converted into the active pheromone compounds [21]. Though *trans*-unsaturated pheromone components are not utilized by *L. capitella*, minor amounts of E11–14:Me were detected in DMDS analyses of FAMEs from female abdominal tips. This was not confirmed *in vitro*, which might be due to the limits of detection and does not alter the above evidences of an active Δ11-gene.

After supplementation with excess of Z9–14:Me, the yeast produced small amounts of the conjugated Z9,Z11-tetradecadienoate, which was detected upon GC-MS analyses of the *cis *MTAD-adduct (Fig. 5B). In presence of large amounts of both pheromone biosynthetic intermediates (*i.e*., the 16:acyl and the Z9–14:acyl), the *Lca*-KPVQ transcript always preferentially catalyzed the formation of the Z11–16:acyl monounsaturated intermediate.

Δ9-stearyl-CoA desaturases are primordial enzymes regulating the level of unsaturated fatty-acid biosynthesis and thereby allowing living organisms to maintain the physical structure and fluidity of membrane lipid bilayers [42-44]. When transforming yeasts with YEpOLEX-*Lca*-SPVE or YEpOLEX-*Lca*-GATD constructs, the Δ9 desaturase activity of the *ole1 *yeast strain could not be restored. Δ9-desaturases (18C>16C) exist that catalyse the formation of Z9-monoenes in the pheromone gland of some ditrysian moth species [19-21,23,25]; although in most cases pheromone biosynthesis does not involve Δ9 desaturation [10]. It is also not uncommon to encounter inactive desaturase transcripts as exemplified by two *Lca*-GATD paralogs, the *Hass*GATD transcript that was found to be non-functional in pheromone biosynthesis in *H. assulta *[26] or the *Cpa*Z9GATD (C14-C26) transcript that produced a long series of monounsaturated fatty acids in *C. parallela*, an activity that could however not be linked to sex-pheromone biosynthesis [25]. It might then be envisioned that both *Lca*-SPVE and *Lca*-GATD are catalytically inactive desaturases in this moth species and as previously suggested, could represent pseudogenes or encode other functions yet to be determined [10,24,28]. Analyses of esterified total-lipid extracts of YEpOLEX-*Lca*-QPAQ-transformed yeast revealed that the gene product was producing a series of Z9-monoenes with a preference for palmitic acid like other Δ9 16C>18C insect-desaturases (Fig. 4). The Z9–14:acyl, intermediate in the pheromone biosynthesis (pathway ***b***, Fig. 6), accounted for 2% of the total amount of UFAs produced and could thus be formed by the *Lca*-Z9-QPAQ desaturase. However, topical application of labelled precursors indicated that the D_9_-Z11–16:acyl is also significantly incorporated into Z9–14:acyl. Knowing that the Z11–16:acyl is produced by Δ11-desaturation and that significant chain-shortening occurs in the gland, it is more likely that the Z9–14:acyl derives from β-oxidation of the Z11–16:acyl precursor. The *Lca*-Z9-QPAQ desaturase is most likely not involved in the pathway ***b ***of pheromone production. Besides, no conjugated Z9,Z11–14:acyl could be detected from *Lca*-Z9-QPAQ yeasts transformants grown in presence of Z11–14:Me and this transcript is therefore not involved in the Δ9-desaturation reaction proposed in the pathway ***c ***(Fig. 6).

In conclusion, our *in vitro *analyses indicate that the *Lca*-Z11-KPVQ transcript encodes a functional protein displaying enzymatic properties consistent with the proposed pheromone biosynthetic pathway of *L. capitella *(pathway ***a***, Fig. 6). While the first Z11-desaturation step on palmitic acid is a prevalent reaction that has been characterized in several other ditrysian moth species (see *e.g*., [17,26,27]), the Δ11-desaturase activity on monoene precursors has been evidenced in only a few moth species up to date. In *E. postvittana*, an E11-desaturase catalyses the formation of E9,E11–14:acid from E9–14:acid [24] while in *B. mori*, *Manduca sexta *and *S. littoralis *bifunctional Δ11 and Δ10,12-desaturases catalyse the formation of conjugated Δ10,12 dienes from Z11-monounsaturated precursors [27,29,31].

### Insights into the origin of the Δ11-desaturase subfamily

Any gene in any genome is selectively constrained and most mutations that change the fitness of an organism are expected to be deleterious [45-47]. Gene duplications represent major opportunities that contribute to functional novelties in all living organisms thereby playing a vital role during evolution [47,48] and the predominant motive during the evolution of a novel gene function is to gain a selective advantage [45]. The need for efficient mate signalling and species-specific recognition might have been a strong motive for establishment of new functions within pheromone communication as derived from duplicated genes. The pheromone-desaturase family most likely originated in a common ancestor of Diptera and Lepidoptera before their divergence in the early Carboniferous, *i.e*., between 330 and 350 MYA [49]. Several extant flies hence use orthologs of the Δ9 (16C>18C) group (Fig. 4)-which represents the metabolic ancestral function of the gene family [10,15]- for synthesizing cuticular-hydrocarbon sex-pheromones [9,50]. Subsequently the lepidopteran desaturase gene family evolved under a birth-and-death evolutionary process and underwent several duplication events leading to five well-supported clades [10,21,51] (Fig. 4). Reconstructing the evolutionary history of desaturase genes shows that only the Δ11 and the Δ9 (18C>16C) lineages have no orthologous genes in any Dipteran genome examined to date (Fig. 4), which suggests that the genes were either lost in Diptera subsequent to the divergence of moths and flies or that both lineages may have been recruited following a duplication point succeeding the divergence between Diptera and Lepidoptera [10,21]. We have examined the evolution of desaturases using predicted genes available from other insect orders including Hymenoptera, Hemiptera and Coleoptera (see additional File 1) and we found no evidence for orthologous genes of the Δ11 and Δ9 (18C>16C lineages) in these insect orders. Both desaturase lineages are thus most likely innovations of lepidopterans, of which the Δ11-desaturase lineage exclusively serves in the pheromone production. Our molecular and functional investigations of pheromone biosynthetic genes in *L. capitella *strongly indicate that the duplication events that gave rise to both desaturase-gene lineages took place before the divergence of Heteroneura lineages from Ditrysia, around 125 MYA. The alternative explanation that both duplications occurred independently in two different lineages is a less parsimonious explanation.

Clearly, the emergence and establishment of the Δ11-desaturase subfamily in moth genomes might have promoted structural diversity among pheromones early in the evolution of Lepidoptera. Whether the duplication events took place before or after the divergence of Lepidoptera and its sister group Trichoptera will be interesting to investigate. In this context it would be of interest to find out whether Δ9- or Δ11-desaturases (or another desaturase function yet unidentified) underlie the double-bond formation in the biosynthesis of (*Z*)-4-hepten-2-ol and (*Z*)-6-nonen-2-ol (and their corresponding ketones) as observed in Eriocraniidae moths and in Trichoptera. Both compounds could hypothetically be produced by Δ11-desaturation of tetradecanoate, followed by chain shortening by successive steps of β-oxidation and finally decarboxylation [38]. Investigating pheromone biosynthesis in these more primitive non-ditrysian moth lineages will further highlight to what extent the recruitment of discrete pheromone production genes contributed to the emergence of the typical ditrysian moth signalling chemistry and by extension promoted the adaptive success of the Lepidoptera.

Likewise, the exact location of the distal pheromone-producing cells and their morphological features in Monotrysia should also be investigated. Such an investigation could resolve the intriguing issue whether the pheromone-producing desaturases in monotrysian moths are catalysing the formation of biosynthetic precursors in a pheromone gland structure alike those of most ditrysian species or within -a yet uncharacterized internal structure- of the abdominal tip.

## Conclusion

The renowned sexual communication of Lepidoptera has likely evolved through a combination of mechanisms [2,36] involving the diversification of female-produced chemical signals that mediate mate attraction and corresponding changes in male responses to these signals [52]. Variation in pheromone-biosynthetic genes have been supported for promoting changes in the emitted signals thereby indirectly directing major adaptations in moth mate-recognition systems [53]. A core component of the evolution of moth chemistry has been evidenced that imply gene duplications of biosynthetic genes and their maintenance in the insect genomes [10,15]. We here brought evidence that Lepidoptera-specific pheromone-production desaturase genes evolved before radiation of the ditrysian lineages. Especially, a Δ11-desaturase (*Lca*-KPVQ) was shown catalyzing key reactions leading to mono- and di-unsaturated fatty acyl-moieties in the primitive moth *Lampronia capitella*, the sole non-ditrysian species identified to date that relies on long-chain di-unsaturated fatty-acid derivatives for mate-attraction. Like many other characterized ditrysian-moth desaturases, *Lca*-KPVQ catalyses a few more desaturation reactions *in vitro *than those leading to the biologically relevant components used by *L. capitella*. This supports the current view that subtle alterations in enzymatic activities at different levels in the biosynthesis may have lead to subtle shifts in pheromones and ultimately to new species-specific communication channels [15,21]. Recruiting Δ11-desaturases in mate signalling prior to the extensive lepidopteran radiation may thus have contributed, as part of a complex biological framework, to the evolution of the typical moth pheromone chemistry and supports the hypothesis that a limited number of biosynthetic genes played a pivotal role in ditrysian moth evolution.

## Methods

### Insect collection

Immature larvae were collected in a black currant (*Ribes nigrum*) orchard in mid-May in Sörfors, northwest of Umeå (Västerbotten, Sweden). Infested branches were cut, brought to the laboratory and kept at 15°C until the larvae had pupated. Pupae were separated from branches or folded leaves and were maintained at 23°C in 60% relative humidity and in a 17:7 hours light:dark photoperiod.

### Application of labelled precursors

Virgin 0 to 2 day-old female moths were used for topical application of labelled precursors onto the abdominal tip. In the early photophase, individual calling females were anaesthetized using carbon dioxide and the abdomen was then gently squeezed in order to completely expose the abdominal tip. D_9_-Z11–16:acid (4 μg in 0.2 μl DMSO) was topically applied onto the abdominal tip and the solution was allowed to be absorbed by the gland during a few minutes. Females were reintroduced into individual cages for 24 hours. Abdominal tips from 10 females (including ovipositor) were then carefully dissected under a stereomicroscope and extraction of pheromone components was performed for 1 hour in a glass capillary containing 80 μl hexane.

### Base methanolysis

For analysis of fatty-acid methyl esters, a total lipid extraction was performed using chloroform:methanol (2:1 v/v). Base methanolysis was then utilized to convert fatty-acyl moieties into the corresponding methyl esters. Abdominal tip tissues were removed and the solvent was evaporated under a gentle N_2 _stream. Concentrated extracts were treated with 100 μl 0.5 M KOH/methanol and allowed to react for 1 hour at room temperature. For base methanolysis of total lipid yeast extracts, yeast residues were directly treated with 500 μl 0.5 M KOH/methanol after solvent evaporation [17]. Abdominal tip samples or yeast extracts were acidified by addition of 100 or 500 μl 1.0 M HCl, respectively and the resulting fatty-acid methyl esters (FAME) were collected in hexane, and analysed by GC-MS analysis.

### Determination of double bond positions by DMDS and MTAD

Methanolyzed extracts were converted into dimethyl-disulfide (DMDS) derivatives by addition of 50 μl DMDS and 5 μl iodine 5% in diethyl ether and then incubated over night at 40°C. Two hundred μl of hexane were added to the sample and the reaction was neutralized by addition of 20 to 50 μl Na_2_S_2_O_3 _5% [54]. The organic phase was removed, concentrated under a gentle N_2 _stream and subjected to GC-MS analysis. The 4-methyl-1,2,4-triazoline-3,5-dione (MTAD) adducts were prepared by transferring 5 μl of the methyl ester extracts into a glass vial containing 10 μl CH_2_Cl_2 _and treating the resulting solution with 10 μl of a MTAD solution (2 μg/μl; in CH_2_Cl_2_). Reactions were incubated for 15 min at room temperature and 2 μl were subjected to GC-MS analysis [55].

### Gas chromatography-mass spectrometry analyses

Samples were analysed on a gas chromatograph (GC) (Hewlett Packard HP 6890 GC system) equipped with an auto-injector (HP 7683) and coupled to a mass selective detector (HP 5973). The GC was equipped with an HP1-MS column (100% methyl siloxane; 30 m 3 0.25 mm, d_f_: 0.25 μm) and helium was used as carrier gas (velocity: 32 cm/s). For FAME and DMDS analyses, the oven temperature was set to 55°C (or 80°C) for 2 min, then increased by 10°C/min up to 250°C, followed by a hold at 250°C for 10 min, and then increased by 20°C/min up to 300°C followed by a hold at 300°C for 5 min. For MTAD adduct analyses, the injector temperature was set to 300°C, the oven temperature was set to 100°C and then increased by 15°C/min up to 300°C followed by a hold at 300°C for 20 min according to procedures previously described [55].

### FAME synthesis

(11*Z*)-Tetradeca-11-en-1-ol and (9*Z*,11*E*)- and (9*Z*,11*Z*)-tetradeca-9,11-dien-1-ols (12–115 mg), respectively, were oxidised at 0°C with Jones reagent according to [56] followed by acid-catalysed esterification of the resulting acid in methanol at room temperature.

### Total RNA isolation and cDNA synthesis

Abdominal tips were carefully dissected from 50 virgin female moths on the first day after their emergence, immediately snap frozen in liquid nitrogen and stored at -80°C. Total RNA was isolated and purified from abdominal tips using the Trizol^® ^reagent (Invitrogen™ Life technologies) according to recommended procedures. One μg of total RNA was reverse transcribed into cDNA using Stratascript (Stratagene) following the manufacturer's protocol.

### PCR screening and cloning of pheromone desaturase-encoding cDNAs

Abdominal tip cDNA was used as template for PCR amplification using the primers PR3 and PR4 (Table 1) designed based on the highly conserved GAHR and EGFH histidine-rich motifs identified in other insect desaturases [21]. PCR reactions were performed in a PCR GeneAmp 9700 Thermo Cycler (Applied Biosystems) using the AmpliTaq Golds chemistry (Applied Biosystems). The following temperatures were used for cycling: 94°C for 4 min followed by 30 cycles at 94°C for 30 s, 50°C for 30 s and 72°C for 1 min 30 s followed by a final step at 72°C for 40 min, to ensure complete extension of all products to maximize TA cloning. Amplification products were analysed by electrophoresis on 1.5% agarose gels and visualized with ethidium bromide. Expected 550 bp-amplification products were excised from the gel, purified using the Qiagen gel extraction kit (Qiagen) and then ligated to TOPO^® ^TA PCR 2.1 vector (Invitrogen). The ligation mixtures were used to transform *E. coli *TOPO 10 competent cells (Invitrogen). Plasmids were purified using standard protocols and plasmid DNAs were subjected to sequencing reaction using the Big Dye Terminator cycle sequencing kit v1.1 (Applied Biosystems) followed by analysis on a capillary ABI 3100 sequencer instrument (Applied Biosystems). Sequence information was obtained in both forward and reverse directions and the curated consensus sequences were used for database searches to verify the identity of the isolated central gene fragments. Based on the central sequence information, gene-specific primers (GSPs, Table 1) were designed to PCR-amplify the 3'-and the 5'-cDNA terminal regions using the RACE SMART Kit (Clontech). PCR amplifications were performed according to the manufacturer's instructions and GSPs used for a first and a second round of PCR are listed in Table 1. The 5'and 3' PCR products were cloned and sequenced as described above. The entire DNA sequence for all desaturase genes were compiled and GSPs (Table 1) were designed for the PCR amplification of the entire DNA region representing each gene. Obtained PCR products were cloned and sequenced to verify the integrity of each desaturase gene.

**Table 1:** Oligonucleotide primers used for desaturase gene characterization and functional expression

Degenerated	Primer sequence^a^
PR3	5'-GGYATYACVGCHGGNGCWCA-3'
PR4	5'-TGRTARTTRTGGAABSCYTCNCC-3'

**Gene specific^b^**	**Primer sequence**

Lc1–5a	5'-CAGGCCAGCGGCATCAGCAATAAGTA-3'
Lc1–5b	5'-CGAGGATTAATCTCAGCGGCCACT-3'
Lc1–3a	5'-TACTTATTGCTGATGCCGCTGGCCTGC-3'
Lc1–3b	5'-TCCTGGAAAAACGCGCTCTTCGTAGCTGCAATG-3'
Lc2–5a	5'-TCCTTTGGCCTTGATTTCGGGGTGTTTC-3'
Lc2–5b	5'-CCATGAGCCAACCCATGTGGGAGAAGAA-3'
Lc2–3a	5'-GGTGAAGAAACACCCCGAAATCAAGGCC-3'
Lc3–5a	5'-TCGCTCATGTCAATGCTGGCTCCCTTCT-3'
Lc3–5b	5'-ACGTTTGGCGTTGTGAGGATCAGCGTC-3'
Lc3–3a	5'-TTGAGGCGGACCCCATCGTCATGTTT-3'
Lc3–3b	5'-TGGTACGTGGCTACAATCCTGCGGTTCA-3'
Lc4–5a	5'-GTCAACAGCCAACCGACATGCGAA-3'
Lc4–5b	5'-GAGTCGGGATAACGAAGCAAACCA-3'
Lc4–3a	5'-TACGCAATTCCCTTCGTCGGATTG-3'
Lc4–3b	5'-CTTATGCCGATGTACTTCTGGAACG-3'

**Full-length amplification^b^**	**Primer sequence**

Lc1-fls	5'-GATTCATAGATTCGTGTTCGGTGA-3'
Lc1-flas	5'-CAGGGACCTCGAAGTGACCTTT-3'
Lc2-fls	5'-GCAGTGATTGGTGTCGTGCGGA-3'
Lc2-flas	5'-AACAATAAAATATTTATTTACATTAATTTGG-3'
Lc3-fls	5'-TAAAGCAAGTTACTGTCGCCG-3'
Lc3-flas	5'-GAAATACGTTTATTATTGGAATAAATAA-3'
Lc4-fls	5'-GAGTCACAAGTTGTTCAGTCGTAA-3'
Lc4-flas	5'-TCCATAAGATGGGTACGTATATTGT-3'

**Expression YEpOLEX**	**Primer sequence^c^**

Lc1ORFs	5'-gctctaga**ATG**CCGCCGAACGTGACAG-3'
Lc1ORFas	5'-cgagctcg**CTA**ATCATCTTTACGGTTAATG-3'
Lc2ORFs	5'-gctctaga**ATG**CCGCCCGAGGGTCTAAT-3'
Lc2ORFas	5'-cgagctcg**TCA**AAAAGATTTCTCCGGAT-3'
Lc3ORFs	5'-gctctaga**ATG**GCTCCAAACATCCTGGG-3'
Lc3ORFas	5'-cgagctcg**TCA**CTGTTCTTTAGGATGTGC-3'

**Expression pYEX**	**Primer sequence^c^**

Lc4ORFs	5'-ggatccATA**ATG**GCGCCATATCCTGAAG-3'
Lc4ORFas	5'-gaattcCGATTTCATATTAGTTCATT**TTA**ATC-3'

^a ^[21]; Y = C or T; V = A, G or C; H = A, C or T; N = A, G, C or T; W = A or T; R = A or G; B = G, T or C; S = G or C.

^b ^Lc1= *Lca*-QPAQ, Lc2 = *Lca*-SPVE, Lc3 = *Lca*-GATD, Lc4= *Lca*-KPVQ

^c ^Restriction sites are indicated in lower case letters, start and stop codons are marked in bold.

DNA sequences were analysed using Sequencher V.3.0 software (Gene Codes Corporation, MI, USA) and the Bioedit Sequence alignment editor program V. 5.0.9 [57].

### Sequence analyses and Neighbour-Joining tree construction

Comparison of sequence information to publicly available DNA information was performed using BLAST [58]. Multiple sequence alignments were performed using the Clustal W 18.2 algorithm [59] and edited in BOXSHADE [60]. Prediction of the endoplasmic retention signal was performed using PSORT [61]. Desaturase aa sequences used for phylogenetic reconstructions were retrieved from the GenBank non-redundant (nr) protein database [62] and the Neighbour-Joining tree was constructed using MEGA version 3.1 [63].

### Functional assay of Δ9-desaturases by complementation in mutant yeast using the expression vector YEpOLEX

Gene-specific primers including *XbaI *and *SacI *restriction sites (Table 1) were designed based on the ORFs identified for each of the three Δ9-like desaturase transcripts. After PCR amplification from abdominal tip cDNA, PCR products were purified by standard procedures followed by trimming by double restriction digestion using *XbaI *and *SacI*. Digestion products were purified by gel electrophoresis followed by gel elution and then directionally inserted by replacement cloning in the linearized YEpOLEX plasmid [17]. After verification by DNA sequencing, the final recombinant plasmids were designated YEpOLEX-*Lca*-QPAQ, YEpOLEX-*Lca*-SPVE and YEpOLEX-*Lca*-GATD and used for transformation of a desaturase-deficient yeast strain (*MATα ole1::LEU2 leu2–3 leu2–112 trp1-1 ura3–52 his4*) [12]. The YEpOLEX-*Hass*-KPSE plasmid construct from *Helicoverpa assulta *[26] was used as a positive control. For selection of uracil prototrophs, transformed yeasts were plated on selective medium containing 0.7% YNB and a drop-out medium lacking uracil and leucine (ForMedium™), 2% glucose, 0.5 mM palmitoleic acid, 0.5 mM oleic acid (Larodan, Sweden), 1% tergitol (type Nonidet NP-40, Sigma) and 0.01% adenine (Sigma). Individual uracil prototroph colonies were transferred to YPAD plates for complementation of the UFA auxotrophy of the *ole1 *yeast strain used. UFA prototrophs were inoculated in 10 ml YPAD medium with or without Z11–14:Me and incubated at 30°C and 300 rpm for 48 hours. Cells were collected by centrifugation and cell pellets were washed with sterile water three times and then processed for FAME analyses. The double-bond positions of FAMEs were determined by GC-MS analysis of DMDS adducts.

### Functional assay of a Δ11-desaturase by complementation in mutant yeast using the expression vector pYEX-CHT

The pYEX-CHT expression vector [39] was used for functional assay in combination with the double deficient *ole1 elo1 S. cerevisiae *strain (*MATa elo1*::*HIS3 ole1*::*LEU2 ade2 his3 leu2 ura3*), defective in both desaturase and elongase gene functions [40]. This expression vector is derived from the pYEX plasmid (ClonTech), and is related to the vector used for assaying the biochemical activity of the Δ11-desaturase in *S. littoralis *and *Thaumetopoea pityocampa *[29,30]. Two gene-specific primers, Lc4ORFs and Lc4ORFas (Table 1) encompassing the *BamH1 *and *EcoR1 *restriction sites were designed to amplify *Lca*-KPVQ ORF using the Advantage^®^2 PCR enzyme system (Clontech), which was then ligated into the TOPO^® ^TA PCR 2.1 vector (Invitrogen) and the construct was transformed into TOPO 10 cells. The insert was amplified, purified, released from plasmid by *BamH1 *and *EcoR1 *restriction digestion and subcloned into the *BamH1 *and *EcoR1 *cloning sites of the linearized pYEX vector. Constructs were transformed into TOPO 10 cells and analysed by sequencing. The final construct designated pYEX-*Lca*-KPVQ was transformed for functional expression into the *ole1 elo1 *yeast. pYEX-*Lca*-KPVQ yeast transformants were grown on selective medium as described above for 4 days at 30°C. Individual transformants were then isolated and inoculated in 10 ml YPAD medium containing 1 mM CuSO_4 _and grown for 48 hours at 30°C and 300 rpm. Cells were harvested by centrifugation and then suspended in fresh YPAD medium containing 1 mM CuSO_4 _with or without addition of Z9–14:Me (Larodan, Sweden). The cells were subsequently incubated for 48 hours, collected by centrifugation and then washed with water. Fatty-acid methyl esters were extracted and analysed by GC-MS. The position of double bonds in monoenes and dienes was determined by DMDS and MTAD derivatizations, followed by GC-MS analysis.

## Abbreviations

aa: amino acid; bp: base pair; DMDS: dimethyl disulfide; DMSO: dimethyl sulfoxide; GC-MS: gas chromatography coupled to mass spectrometry; FAME: fatty-acid methyl ester; Lca: Lampronia capitella; MTAD: 4-methyl-1,2,4-triazoline-3,5-dione; MYA: million years ago; ORF: open reading frame; PCR: polymerase chain reaction; RACE: rapid amplification of cDNA ends; TIC: total ion current; UFA: unsaturated fatty acid; YNB: yeast nitrogen base; YPAD: yeast/peptone/adenine/dextrose; Z9,Z11–14:OH: (*Z,Z*)-9,11-tetradecadien-1-ol; Z9–14:acid: (*Z*)-9-tetradecenoic acid; Z11–14:acid: (*Z*)-11-tetradecenoic acid; E11–14:acid: (*E*)-11-tetradecenoic acid; Z9–16:acid: (*Z*)-9-hexadecenoic acid (palmitoleic acid); Z11–16:acid: (*Z*)-11-hexadecenoic acid; Z9–18:acid: (*Z*)-9-octadecenoic acid (oleic acid); Z11–18:acid: (*Z*)-11-octadecenoic acid; Z9,Z11–14:acid: (*Z,Z*)-9,11-tetradecadienoic acid; Z9,E11–14:acid: (*Z,E*)-9,11-tetradecadienoic acid (fatty acyls and FAMEs are named correspondingly).

## Data deposition

The cDNA-sequence information reported in this study has been deposited and are available by the GenBank accession numbers EU152332 to EU152335.

## Authors' contributions

CL conceived the study. TJ and ML participated in its design and coordination. ML carried out the experimental part and compiled the data. MS participated in the desaturase screening and EH synthesized methyl esters for the functional assay. ML and CL wrote the paper with contributions from TJ. All authors read and approved the final manuscript.

## Supplementary Material

Additional file 1 **Phylogeny of desaturase genes of various insect orders**. The Neighbour-Joining tree was constructed using deduced aa sequences and the JTT algorithm (MEGA 3.1, [63]); numbers along branches indicate bootstrap support from 1,500 replicates. Only species for which complete cDNA sequence information or predicted genomic sequences were available (extracted from GenBank) were used. Accession numbers are indicated along the species name. Coloured boxes refer to the different lepidopteran desaturase lineages for comparison with Fig 4. The tree was rooted using the aa desaturase sequence from the tick *Amblyomma americanum*. Click here for file
