# Supplementary figures and images for: Key biosynthetic gene subfamily recruited for pheromone production prior to the extensive radiation of Lepidoptera
Source: BMC Evol Biol. 2008 Oct 2;8:270. doi: 10.1186/1471-2148-8-270 (PMC2584044; doi:10.1186/1471-2148-8-270)

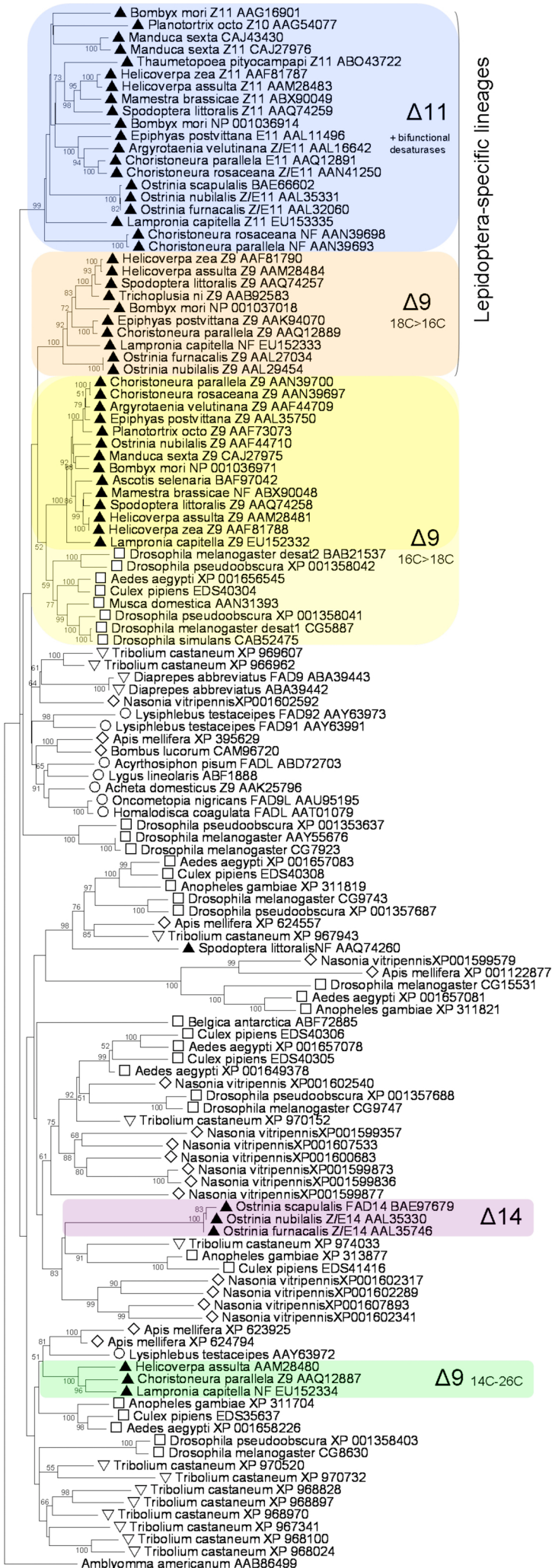

$\Delta 11$   
+ bifunctional  
desaturases

$\Delta 9$   
18C>16C

$\Delta 9$   
16C>18C

$\Delta 14$

$\Delta 9$  14C-26C

Supplement: Additional file 1 — Phylogeny of desaturase genes of various insect orders. The Neighbour-Joining tree was constructed using deduced aa sequences and the JTT algorithm (MEGA 3.1, [63]); numbers along branches indicate bootstrap support from 1,500 replicates. Only species for which complete cDNA sequence information or predicted genomic sequences were available (extracted from GenBank) were used. Accession numbers are indicated along the species name. Coloured boxes refer to the different lepidopteran desaturase lineages for comparison with Fig 4. The tree was rooted using the aa desaturase sequence from the tick Amblyomma americanum. [file 1471-2148-8-270-S1.pdf]
